# The Practical Significance of Measurement Error in Pulmonary Function Testing Conducted in Research Settings

**DOI:** 10.1111/risa.13315

**Published:** 2019-06-03

**Authors:** Richard B. Belzer, R. Jeffrey Lewis

**Affiliations:** ^1^ Mount Vernon VA USA; ^2^ Exxon Mobil Biomedical Sciences, Inc. Clinton NJ USA

**Keywords:** FEV_1_, information quality, intertest variability, intratest variability, measurement error

## Abstract

Conventional spirometry produces measurement error by using repeatability criteria (RC) to discard acceptable data and terminating tests early when RC are met. These practices also implicitly assume that there is no variation across maneuvers within each test. This has implications for air pollution regulations that rely on pulmonary function tests to determine adverse effects or set standards. We perform a Monte Carlo simulation of 20,902 tests of forced expiratory volume in 1 second (FEV_1_), each with eight maneuvers, for an individual with empirically obtained, plausibly normal pulmonary function. Default coefficients of variation for inter‐ and intratest variability (3% and 6%, respectively) are employed. Measurement error is defined as the difference between results from the conventional protocol and an unconstrained, eight‐maneuver alternative. In the default model, average measurement error is shown to be ∼5%. The minimum difference necessary for statistical significance at *p* < 0.05 for a before/after comparison is shown to be 16%. Meanwhile, the U.S. Environmental Protection Agency has deemed single‐digit percentage decrements in FEV_1_ sufficient to justify more stringent national ambient air quality standards. Sensitivity analysis reveals that results are insensitive to intertest variability but highly sensitive to intratest variability. Halving the latter to 3% reduces measurement error by 55%. Increasing it to 9% or 12% increases measurement error by 65% or 125%, respectively. Within‐day FEV_1_ differences ≤5% among normal subjects are believed to be clinically insignificant. Therefore, many differences reported as statistically significant are likely to be artifactual. Reliable data are needed to estimate intratest variability for the general population, subpopulations of interest, and research samples. Sensitive subpopulations (e.g., chronic obstructive pulmonary disease or COPD patients, asthmatics, children) are likely to have higher intratest variability, making it more difficult to derive valid statistical inferences about differences observed after treatment or exposure.

## 1 PULMONARY FUNCTION DATA AS USED IN FEDERAL AIR POLLUTION REGULATIONS

In its 2008 National Ambient Air Quality Standard (NAAQS) for ozone (O_3_), the U.S. Environmental Protection Agency (USEPA, 2008) relied on a chamber study of 30 healthy, exercising young adults in which a transient group mean decrement in forced expiratory volume in 1 second (FEV_1_) of 2.9% was observed after 6.6 hours exposure to 60 ppb O_3_ (Adams, 2006a). USEPA was particularly concerned that two subjects experienced transient FEV_1_ decrements >10% (Brown, 2007). Transient group FEV_1_ decrements ∼5% and transient individual FEV_1_ decrements >10% were deemed to be important for defining adverse effects and for setting national regulations.

Prior to the 2015 O_3_ NAAQS, a new chamber study of 31 healthy young adults was performed in which statistically significant transient group FEV_1_ decrements were observed for at least one exposure duration at 70 ppb, 80 ppb, and 87 ppb, but not at 60 ppb. In percentage terms, the largest group mean decrements were 5% (70 ppb), 7% (80 ppb), and 11% (87 ppb); some subjects experienced decrements >10% (Schelegle, Morales, Walby, Marion, & Allen, 2009). Based on this study, USEPA concluded that “the results of controlled human exposure studies strongly support setting the level of a revised O_3_ standard no higher than 70 ppb” (USEPA, 2015, p. 65353).

Thus, federal air pollution policy considers transient FEV_1_ decrements that exceed 10%, or are determined to be statistically significant regardless of magnitude, as convincing evidence of adverse health effects. It is therefore important to explore whether the test methods used to measure these decrements are appropriate and reliable for that purpose.

## 2 SPIROMETRIC PROTOCOLS

Spirometry in air pollution research settings follows protocols established by the American Thoracic Society (ATS) or the European Respiratory Society (ERS) for use in clinical settings (ATS, 1979, 1987, 1995; Miller, Hankinson et al., 2005; National Health and Nutrition Examination Survey [NHANES], 2008). A “maneuver” is performed when a subject inhales deeply and blows hard into a tube connected to a spirometer. Peak expiratory flow rate (PEF), FEV_1_, forced vital capacity (FVC), and other measurements are calculated. Maximum subject performance is desired but constrained by examiner skill (Enright, Beck, & Sherrill, 2004); subjects’ ability, posture, and cooperativeness; physical setting; season; time of day; and a host of other factors (ATS, 1979, 1987, 1995; Miller, Crapo et al., 2005; Redlich et al., 2014; Stocks, Kirkby, & Lum, 2014), including inherent uncertainty and variability. Tests may be quality graded A through F (Enright et al., 2004; Enright, Johnson, Connett, Voelker, & Buist, 1991; Enright, Skloot, Cox‐Ganser, Udasin, & Herbert, 2010).

The ATS protocol requires that three to eight maneuvers be performed for each test. The average is unlikely to change appreciably after three maneuvers, but the maximum will increase until gains from practice (Enright, 2003) are outweighed by subject fatigue (Miller, Hankinson et al., 2005). For a specific patient in a clinical setting, this appears to be sufficient. For air pollution research, however, eight maneuvers may not be optimal; improvement has been shown after the eighth maneuver (Lehmann, Vollset, Nygaard, & Gulsvik, 2004), and up to 30 maneuvers may be required to obtain “best” performance in young children (Aurora et al., 2004).

This article quantifies measurement error resulting from unreported or unmeasured within‐subject variation across tests (intertest variability) and, more importantly, within‐subject variation across maneuvers within a single test (called intratest or within‐test (Enright et al., 2011; Kainu, Lindqvist, Sarna, & Sovijärvi, 2008; Kainu, Lindqvist, Sarna, Lundbäck, & Sovijärvi, 2008), within‐occasion (Aurora et al., 2004; Beydon et al., 2007a), intrasession (Kainu, 2008; Kainu, Lindqvist, Sarna, & Sovijärvi, 2008; Kainu, Lindqvist, Sarna, Lundbäck et al., 2008), or intrameasurement repeatability (Beydon et al., 2007a)). Measurement error is shown to be within the bounds of what clinicians consider not biologically meaningful but greater than performance decrements that air pollution researchers report as statistically significant.

### 2.1 Repeatability Criteria and Early Test Termination

Some maneuvers are discarded due to technical deficiencies; only acceptable maneuvers meeting repeatability criteria (RC) are retained and potentially reported (Miller, Hankinson et al., 2005). The stated purpose of RC is to “improve confidence in the diagnostic discrimination of the test and the confidence in which changes in lung function may be interpreted by the physician” (Enright et al., 2004, p. 236). Maneuvers are deemed “repeatable” if the difference between the highest and second‐highest FVC and FEV_1_ are within the RC. If differences exceed the RC, up to eight maneuvers are performed until the RC are met.

RC have been a feature of ATS/ERS protocols since 1979, though the choice of RC has changed. It was set at 0.10 L/sec in 1979 (ATS, 1979), retained there in 1987 after a review (ATS, 1987), widened to 0.20 L/sec in 1995 (ATS, 1995), and narrowed to 0.15 L/sec in 2005 (Miller, Hankinson et al., 2005). There are no objective standards for choosing the “right” RC.

ATS/ERS guidance says within‐day differences in FEV_1_ ≤ 5%, week‐to‐week differences ≤11%, and year‐to‐year differences ≤15% for normal subjects should not be interpreted as clinically meaningful; greater differences apply to chronic obstructive pulmonary disease (or COPD) patients (within‐day ≤13%; week‐to‐week ≤20%) (Pellegrino, Viegi et al., 2005). Similar interpretative guidance has been published for occupational spirometry (American College of Occupational and Envirionmental Physicians, 2016; Redlich et al., 2014). How these thresholds were determined is not reported, and while it is plausible that they implicitly account for intertest variability, there is no basis for inferring that they also account for intratest variability (Hnizdo et al., 2007).

Spirometric protocols also include provisions for early test termination once RC have been met (ATS, 1979, 1987, 1995; Miller, Hankinson et al., 2005). This practice results in the failure to collect readily available, acceptable data, and the retention of “maximum” values that often are not unconstrained test maxima. Even if three maneuvers are sufficient to produce a pair of values satisfying the RC, more maneuvers often produce additional such pairs, some of which may have maxima greater than the maximum of the initial qualifying pair that led to test termination. The failure to collect these data may have negligible effects in clinical practice but it is likely to distort air pollution research studies.

### 2.2 Test Failure Due to Lack of Repeatability

Some research subjects cannot perform spirometric tests that are acceptable (i.e., technically valid). Clinical guidance acknowledges these problems and admonishes that the inability to perform spirometry may itself be evidence of lung impairment (Pellegrino, Viegi et al., 2005). Preexisting respiratory conditions (e.g., asthma, chronic obstructive pulmonary disease (COPD)) increase the proportion of subjects who fail (Pellegrino, Decramer et al., 2005).

Subjects may produce acceptable maneuvers but not be able to produce repeatable ones. A retrospective study of 18,000 spirometric tests conducted at the Mayo Clinic indicated that about 95% of patients could produce repeatable maneuvers with  RC =0.20L/ sec , the 1995 criterion. The authors wanted the RC tightened so that only 90% would pass. They acknowledged but dismissed reduced performance observed among subjects who were short, female, or had worse baseline lung function (Enright et al., 2004).

In a large sample of Norwegians, believed to be representative, 12.7% of females and 7.7% of males failed to meet the 1987  RC =0.10L/ sec , and 6.8% of females and 7.1% of males failed under the less demanding 1995  RC =0.20L/ sec  (Langhammer, Johnsen, Gulsvik, Holmen, & Bjermer, 2001). Indeed, it was the disparate effect of the 1987 RC that led to its relaxation in 1995 (ATS, 1995).

A comparison across 14 sites worldwide, each with approximately 600 adult subjects aged ≥40, following the tighter 2005 RC and using identical spirometers with centralized examiner training, had approximately 10% failure rates, but higher failure rates for older subjects (Enright et al., 2011). Similar age‐dependent failure rates in baseline performance have been observed elsewhere (Kainu, Lindqvist, Sarna, Lundbäck et al., 2008; Lehmann et al., 2004). Whether the test failure rate is 5% or 10% in a representative sample, however, the absence of data from these research subjects creates interpretative difficulties. It imparts a form of nonresponse bias for which no statistical adjustment offers a remedy.

### 2.3 Managing Missing Data Due to Failure to Satisfy RC

The same clinical guidance that calls for the application of RC with early test termination also recommends against discarding valid data (ATS, 1979, 1987, 1995; Miller, Hankinson et al., 2005). Other clinical guidance favors the collection of more rather than fewer data and the deletion of *no* data, and criticizes device manufacturers that do not store data from all maneuvers: “[I]t can be tempting to discard any apparently discordant results during data collection before having the chance to inspect them more carefully. This runs the risk of retaining data that are ‘reproducibly wrong’ while discarding physiologically valid results!” (Stocks et al., 2014, p. 173).

ATS/ERS guidance is unclear concerning what the examiner is to do if a repeatable pair cannot be obtained. Examiners are advised that testing should end if “[a] total of eight tests [*sic*; should be “maneuvers”] have been performed (optional) or [t]he patient/subject cannot or should not continue” (Miller, Hankinson et al., 2005, p. 325). However, ATS/ERS also advises that “[n]o spirogram or test result should be rejected solely on the basis of its poor repeatability. The repeatability of results should be considered at the time of interpretation. The use of data from manoeuvres with poor repeatability or failure to meet the [end of test] requirements is left to the discretion of the interpreter” (Miller, Hankinson et al., 2005, p. 326).

In air pollution research studies, the inherent ambiguity in this guideline may lead to inconsistent data collection and reporting. The examiner could (1) discard subjects who cannot produce a highest and second‐highest FVC and FEV_1_ within the bounds of the applicable repeatability criterion; (2) assign the highest values obtained irrespective of whether the repeatability criterion is met; or (3) exercise discretion in some other manner to choose which values to assign to the test. The effects of these alternative approaches to missing data are potentially very different, and they are generally not reproducible.

## 3 WITHIN‐PERSON TEST VARIABILITY

Variation in spirometry is expected due to age, sex, height, and other factors (ATS, 1979, 1987, 1991, 1995; Hnizdo, Glindmeyer, & Petsonk, 2010; Miller, Hankinson et al., 2005). For healthy adult never‐smokers, performance generally peaks in one's late 20s and declines at a rate of 20–30 mL/year (Hnizdo et al., 2010). Inter‐ and intratest variation can be represented by the coefficient of variation, $CV_{m}^{t}$, where *t* indexes tests and *m* indexes maneuvers within test *t*. This can be separated into the two components, $CV^{t}$ and $CV_{m}$. $CV^{t}$ can be accounted for using default adjustments (Miller, Crapo et al., 2005) or statistical models (Redlich et al., 2014). It appears that $CV_{m}$ is implicitly acknowledged nearly everywhere (ATS, 1979, 1987, 1995; Miller, Crapo et al., 2005; Miller, Hankinson et al., 2005) but explicitly accounted for nowhere. In practice, the results of multiple maneuvers across tests and maneuvers are summarized by fixed test values, thus implicitly assuming $CV_{m}^{t}$ (and its components $CV^{t}$ and $CV_{m}$) equal zero.

### 3.1 Within‐Person Intertest Variability, $CV^{t}$

Within‐person differences observed across multiple, identically conducted tests are more likely to be meaningful than simple before/after comparisons. When only two tests are performed, “large variability necessitates relatively large changes to be confident that a significant change has in fact occurred” (Pellegrino, Viegi et al., 2005, p. 962). The 13% threshold below which within‐day differences are believed not to be significant for COPD patients (Pellegrino, Viegi et al., 2005) has been estimated to imply $CV^{t}\approx 6\%$, with lesser percentages (e.g., ≈3% and ≈4%) assumed but not verified to apply to normal subjects (Hnizdo et al., 2010).

Values for $CV^{t}>6\%$ have been obtained in population‐representative samples. For example, $CV^{t}$ was estimated at 13% and 12% for men and women, respectively, in a large random sample of asymptomatic Norwegian never‐smokers aged ≥20, using the 1987 RC (0.10 L/sec). A separate examination of the 19 nurses and technicians who performed the tests yielded a sample mean $CV^{t}$ = 4% (Langhammer et al., 2001). The reason for these differences is not explained, but might be due to greater homogeneity among nurses and technicians, their expertise in spirometry, or both.

In a project intended to identify the index of lung function with the highest signal‐to‐noise ratio (i.e., the highest ratio of between‐ to within‐subject variance), researchers reported mean $CV^{t}$ of 2.7% for FVC and 3.3% for FEV_1_. These estimates were within the range of values reported in previous studies (FVC: 1.8−4.9%; FEV_1_: 2.3−4.7%), but all were unrepresentative small samples, making generalizations inappropriate (Künzli, Ackermann‐Liebrich, Keller, Perruchoud, & Schindler, 1995). $CV^{t}$ values for children also have been reported (Beydon et al., 2007a), but their relevance to adults is unclear, the methods used to obtain them are different, and a larger fraction of children tends to fail spirometric testing (Loeb et al., 2008).

### 3.2 Within‐Person Intratest Variability, $CV_{m}$

In every spirometric study we have reviewed, there appears to be an implicit assumption that $CV_{m}=\;0$. Large‐scale epidemiological studies such as NHANES (2008, 2008, 2011) also implicitly assume $CV_{m}=\;0$ because fixed values are reported for each test. $CV_{m}$ was calculated for each subject in a randomized sample of 648 Finns aged 25−75 (M: 248, F: 355), 603 (93%) of whom met the 1995 RC (0.15 L/sec). Across the sample, mean $CV_{m}$ for FEV_1_ was 1.4% (95% CI = 1.36−1.51). The distribution of subject‐specific $CV_{m}$ values was not reported (Kainu, Lindqvist, Sarna, & Sovijärvi, 2008). The retrospective study of Mayo Clinic spirometry data reported mean $CV_{m}$ for FEV_1_ ranging from 2.65% to 3.35% among males and from 1.9% to 4.1% among females. In both cases, estimated $CV_{m}$ was downwardly biased by the apparent exclusion of subjects who could not meet the RC. Unacceptable and nonrepeatable maneuvers were higher among older subjects and those with diminished health status, and $\;CV_{m}$ increased with smaller physical stature (Enright et al., 2004).

### 3.3 Estimating Intratest Variability in the U.S. Population from NHANES Data Exclusion Rates

The proportion of acceptable maneuvers in the U.S. population excluded due to the RC can be inferred from NHANES (2011). Table I reports the number of maneuvers performed across the sample. If a minimum of three maneuvers is performed, the number of maneuvers should be the same for first through third maneuver. For unexplained reasons, NHANES reports more third than second maneuvers and more second than first maneuvers. The numbers of fourth through eighth maneuvers indicate how many subjects did not meet the RC in maneuvers three to seven, respectively. Three maneuvers of valid data were obtained from approximately 7,200 subjects. However, the RC necessitated a fourth maneuver be performed for 5,035 subjects, 70% of the sample. From maneuver four to maneuver eight, the number of additional maneuvers required ranged from 57% to 71%.

**Table I:** Exclusion Rates in NHANES 2009–2010 Pulmonary Function Testing

Maneuver *m*	Maneuvers Performed ($N_{m}$)	Maneuvers Accepted ($A_{m}^{i}$)^a^	Maneuvers Excluded ($E_{m}^{i}$)^b^	Implied Exclusion Rate ($\%E_{m}^{i}$)^c^
1	6,845^d^		–	–
2	7,169^d^			
3	7,198	2,163	5,035	70%
4	5,035	1,848	3,187	63%
5	3,187	1,136	2,051	64%
6	2,051	687	1,164	57%
7	1,364	394	970	71%
8	970	968	2	0.2%
9	2^e^	–	–	–

a $A_{m}^{i}$ = $N_{m}^{i}$ – $N_{m}^{i+1}$.

b $E_{m}^{i}$ = $N_{m}^{i+1}$.

c $\%E_{m}^{i}$ = $E_{m}^{i}$÷ $N_{m}^{i-1}$.

d Logically, *N*_1_ should always exceed *N*_2_ and *N*_2_ should always exceed *N*_3_. NHANES (2008) provides no explanation why *N*_1_ < *N*_2_ and *N*_2_ < *N*_3_.

e Ninth maneuvers are not documented.

*Source*: NHANES (2008); NHANES uses the ATS (1995) guidelines (three minimum maneuvers; RC = 0.15 L/sec).

Table II shows the data exclusion fractions obtained using the simulation tool described in Section 3 for RC = 0.15 L/sec and $CV_{m}$ values ranging from 1% to 10%. The $CV_{m}$ value that best fits the NHANES data exclusion fractions is about 6%. The NHANES collection was a probability sample, so 6% appears to approximate the average $CV_{m}$ for the U.S. population.

**Table II:** Proportion of Acceptable Results Excluded Due to Repeatability Criterion as a Function of Intratest Variability *CV_m_*

*CV_m_*	Proportion of Acceptable Results Excluded
1%	0.3%
2%	14%
3%	32%
4%	46%
5%	55%
6%	61%
7%	67%
8%	71%
9%	76%
10%	77%

## 4 SIMULATION

To gain insight concerning the effects of RC, $CV^{t}$, and $CV_{m}$, a Monte Carlo analysis was performed for a single subject with defined age, height, and normal pulmonary function. The simulation model assumes that each test, and each maneuver within each test, is statistically independent and earned an A grade. Relaxing these assumptions would only increase inter‐ and intratest variability and strengthen the results reported in Section 3.3.

### 4.1 Default Model

Table III shows default simulation model parameters. Normal pulmonary function was obtained from a specific reference equation (Brändli, Schindler, Künzli, Keller, & Perruchoud, 1996). A $CV^{t}$ of 3% ($\sigma^{t}=\;0.11\;L/\sec$) was obtained from Künzli et al. (2000), and $CV_{m}$ is assumed to be 6% ($\sigma_{m}=\;0.21\;L/\sec$) based on the population value derived from NHANES (2008). To ensure high statistical power (90%) and a low nominal *a priori* Type I error rate (5%), 20,902 tests were performed (Robey & Barcikowski, 1992, Table I). For each independent test, eight independent maneuvers were performed using each test's simulated mean FEV_1_ and an intratest standard deviation of 0.21 (i.e., $CV_{m}=\;6\%$).

**Table III:** Default Simulation Parameters

Parameters	Common to Both Models	
Predicted max FEV_1_ (Brändli et al., 1996)	$e^{-8.240+1,9095\ln\left(H\right)-0.0037\left(A\right)-0.000033\left(A^{2}\right)}=3.55\;\;L/\sec,$ where (*H*)eight = 173 cm and (*A*)ge = 59 years.	
Estimated *SD* FEV_1_ (Brändli et al., 1996)	0.51	
Tests	20,902	
	

Parameters	ATS (1995) Protocol	Unconstrained
Maneuvers/test	Minimum required based on RC	8
Repeatability criterion (RC) (ATS, 1995)	0.15 L/sec	None
Intertest CV ($CV^{t}$) (Hnizdo et al., 2010)	3%	3%
Intratest CV ($CV_{m}$) (Enright et al., 2004)	6%	6%

This procedure assures that intratest variability is accounted for without affecting the simulated value obtained for each test. Maneuvers are not in fact independent, though it is unclear how to model their dependence. Subjects’ performance may improve during early maneuvers due to learning and decline in later maneuvers due to fatigue. Thus, if maximum performance is the desired goal, there is an optimal number of maneuvers. But this optimum is unknown, and it is likely to vary across subjects and over time within subjects.

To replicate the ATS/ERS protocol, the first three simulated maneuvers from each test were examined to determine whether the highest and second‐highest FEV_1_ differed by ≤0.15 L/sec. If such a pair was found, the highest value was deemed the maximum, it was recorded as the fixed representation for that test, and the test was presumed to have been terminated. If no qualifying pair was found, the fourth maneuver was compared to the maximum of the first three maneuvers. If the difference between the fourth maneuver and the maximum of the first three maneuvers was ≤0.15 L/sec, the greater of the two values was deemed to be the test maximum and the test was terminated. This procedure was conducted iteratively for up to eight maneuvers to obtain the ATS/ERS protocol maximum.

In the alternative model, tests were not terminated when the RC were met. The highest value across all eight maneuvers was deemed to be the maximum for each test. The difference between the unrestricted maximum for each test and the deemed ATS/ERS maximum equals the magnitude of measurement error for each test.

### 4.2 Managing Reproducibility Failure

**Figure 1:**
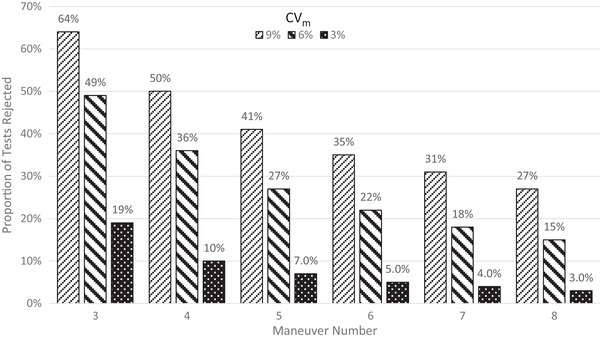
Percentage of simulated tests rejected for lack of ATS/ERS maneuver repeatability, by number of maneuvers performed.

Discarding valid results that do not satisfy the RC reduces the apparent intratest variability by excising the tails of the distribution. The more stringent the RC, the larger will be the tails excised and the degree to which intratest variability is understated. How much understatement occurs depends on the maneuver at which the RC are met and testing terminates. This is shown in Fig. 1. If only three maneuvers are performed, the number of simulated tests that yield no repeatable pair is about 20% at $CV_{m}=\;3\%$, 50% at $CV_{m}=\;6\%$, and 65% at $CV_{m}=\;9\%$. Unless $CV_{m}$ is very low, even the full complement of eight maneuvers may not be enough to produce sufficient data to ensure that the highest feasible maximum is obtained and intratest variability is not materially understated.

As noted in Section 2.3, a choice must be made with respect to the management of simulated tests that fail to produce repeatable pairs and thus cannot be modeled using a strict application of the ATS/ERS protocol. We interpreted the ATS/ERS protocol to require these tests be discarded (option 1 in Section 2.3). Expressed another way, ATS/ERS assumes that subjects who produce maneuvers satisfying the RC are no different from subjects who cannot—an assumption that is likely to be incorrect and artifactually reduce the significance of $CV_{m}$.

Our approach likely departs from typical practice in chamber study and observational epidemiology. In no study in either genre that we have examined have we found subjects excluded for failure to satisfy RC. This means researchers employed options 2 or 3 from Section 2.3, and embedded measurement error may be impossible to estimate. At $CV_{m}=\;6\%$, there is a 15% probability that no repeatable pair will be obtained even after eight maneuvers (see Fig. 1). Thus, 15 of every 100 subjects are expected to be excluded under the ATS/ERS protocol due solely to lack of repeatability even if all eight maneuvers are performed. Further, much larger fractions will be excluded if researchers conduct only three to four maneuvers. This practice, which is clearly undesirable, nevertheless may be necessary in a research design requiring hourly tests (Adams, 2006a, 2006b; Schelegle et al., 2009). The burden of performing eight maneuvers during the last 10 minutes of each hour may be greater than even young, healthy, athletic subjects can tolerate.

### 4.3 Default Model Results

We compare results from the ATS/ERS protocol terminated after three maneuvers with an unrestricted eight‐maneuver model. This comparison maximizes the magnitude of measurement error, but it appears to most closely approximate actual practice in research settings.

**Figure 2:**
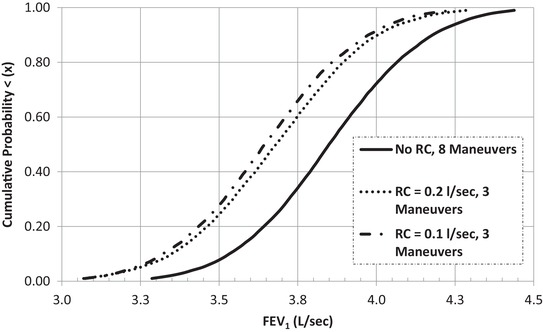
Proportion of FEV_1_ tests without a pair of maneuvers satisfying ATS/ERS reproducibility criterion (*RC*) for three alternative intratest coefficients of variation (*CV_m_*). *Note*:  FE V1t=1 to 20,902 = 3.55 L/sec; CVt=1 to 20,902 = 3%; CVm=1 to 8t= 6%;  RC  range = 0.1−0.2 L/sec.

Fig. 2 compares the cumulative probability density functions for FEV_1_ under the ATS/ERS protocol and the unrestricted eight‐maneuver alternative for RC values ranging from 0.1 to 0.2 L/sec. The horizontal difference is measurement error at each point. It is visually apparent that the cause of measurement error is less the *choice* of RC than the practice of terminating testing once *any* RC are satisfied.

**Figure 3:**
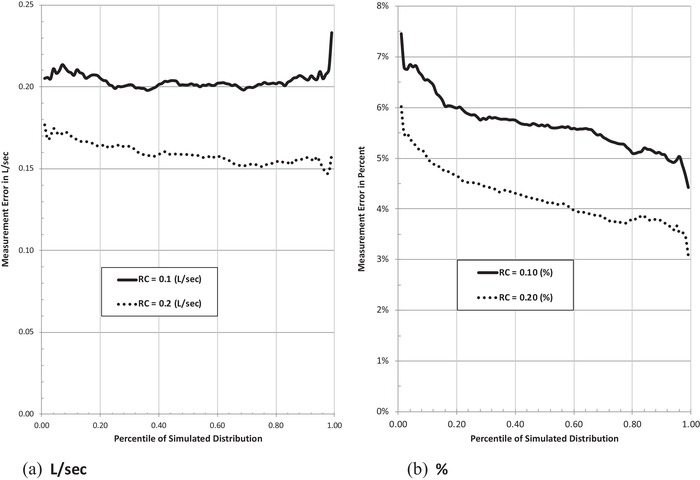
Mean FEV_1_ measurement error resulting from test termination after three maneuvers compared to unconstrained maximum (L/sec and %). *Note*:  FE V1t=1 to 20,902 = 3.55 L/sec; CVt=1 to 20,902 = 6%; CVm=1 to 8t= 6%;  RC  range = 0.1−0.2 L/sec.

Measurement error can be characterized in L/sec or percentage of baseline. This is shown in Figs. 3(a) (L/sec) and (b) (percentage) for the range of RC values considered. Mean measurement error ranges from about 0.15 L/sec (at  RC =0.10L/ sec ) to about 0.20 L/sec (at  RC =0.20L/ sec ), with the difference rising across the simulated distribution. In percentage terms, however, measurement error can exceed 7% and is never less than 3%.

These magnitudes are neither small nor unimportant in air pollution research. They are the same or greater than within‐day differences for normal subjects that are interpreted by clinicians as not meaningful (Pellegrino, Viegi et al., 2005). They are also considerably greater than reported differences in spirometric performance attributable to test‐subject posture (0.04−0.07 L/sec), which ATS occupational guidance deems a confounding factor large enough to warrant preventive control (Redlich et al., 2014).

### 4.4 Minimum Differences Required to Infer that FEV_1_ Pairs Come from Different Distributions

**Figure 4:**
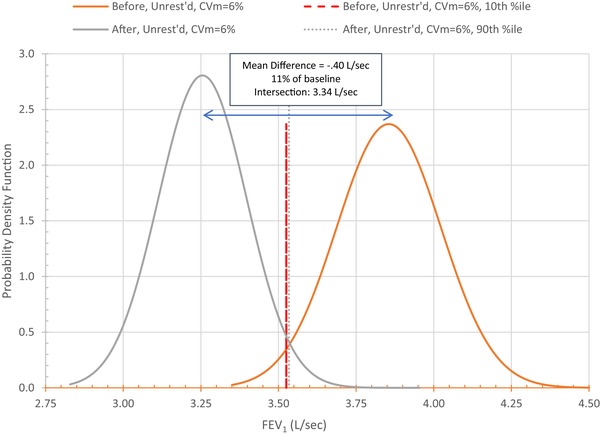
Minimum decline in FEV_1_ necessary for statistical significance at *p* ≤ 0.05 taking only $CV^{t}$ into account.

**Figure 5:**
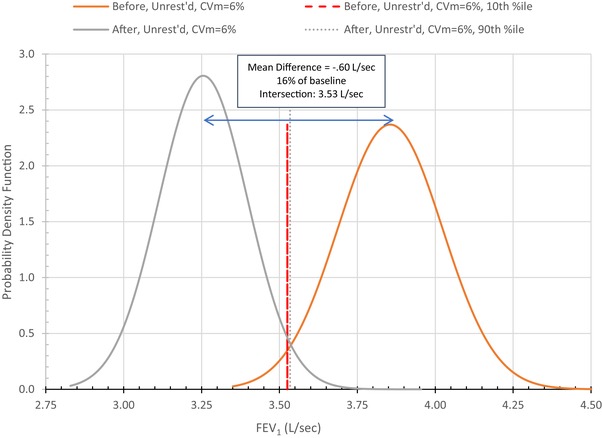
Minimum decline in FEV_1_ necessary for statistical significance at *p* ≤ 0.05 taking both $CV^{t}$ and $CV_{m}$ into account.

Conventional practice treats each test result as fixed (i.e., the intertest standard deviation, $\sigma^{t}$, equals zero), so all differences across tests, no matter their magnitude, are treated as presumptively meaningful. Accounting for inter‐ and intratest variability requires differences to be examined statistically. For example, taking only intertest variability into account, the default model assumes $\sigma^{t}$ = 0.11 (derived from $CV^{t}=\;3\%)$. Fig. 4 shows that any pair of FEV_1_ values must differ by ≥0.4 L/sec (11%) to infer at *p* ≤ 0.10 that they come from different distributions (e.g., before and after exposure). When both inter‐ and intratest variability are accounted for, this difference must be ≥0.6 L/sec (16%). This is shown in Fig. 5, which juxtaposes on the same scale the pre‐ and postexposure distributions necessary for (1) the postexposure FEV_1_ to be below the 10th percentile of the preexposure distribution, and (2) preexposure FEV_1_ to be above the 90th percentile of the postexposure distribution. The gap between these FEV_1_ values—0.6 L/sec—is the minimum difference between pre‐ and postexposure mean FEV_1_ for differences to be statistically significant at *p* < 0.10. (Stipulating that FEV_1_ is reasonably expected to decline after exposure, this is equivalent to a one‐tailed test at *p* < 0.05.)

### 4.5 Sensitivity Analysis

The simulation model allows results to be calculated using alternative values for the subject characteristics such as sex, height, and age; the ATS/ERS protocol attribute RC; and measures of within‐person inter‐ and intratest variability $CV^{t}$ and $CV_{m}$. Subject characteristics matter because the RC, a constant, is a rising fraction for persons with lower FEV_1_ due to age or short stature. For these persons, a larger fraction of valid maneuvers will fail to satisfy the RC. However, the higher rejection rate is counteracted by the ATS/ERS requirement to conduct additional maneuvers, which, *ceteris paribus*, results in higher maximum test values. If researchers strictly follow the ATS/ERS guidelines and collect up to eight maneuvers, the disproportionate effect of the fixed RC on subjects whose normal pulmonary function is below average will be attenuated. However, they will still have a substantial fraction of subjects for whom there is no acceptable pair of maneuvers, as shown in Table II, and no objective path to resolution.

Sensitivity analysis of intertest variability shows that it has a minimal effect regardless of model. However, differences in intratest variability have substantial effects. These differences are summarized in Table IV across the range of ATS/ERS RC values for the default $CV_{m}$ (6%) and two alternatives on either side (0% and 3%, 9%, and 12%). Halving the default $CV_{m}$ reduces measurement error by about 55%. Increasing the default $CV_{m}$ by half increases measurement error by about 65%, and doubling the default $CV_{m}$ increases measurement error by about 125%. $CV_{m}=\;0\%$ corresponds to the ATS/ERS protocol, which by assuming no intratest variability implies no measurement error.

**Table IV:** Mean FEV_1_ Measurement Error Under ATS/ERS Protocol After Three Maneuvers, by Repeatability Criterion (RC) and Intratest Coefficients of Variation ($CV_{m}$) (L/sec and %)

	Intratest Coefficient of Variation ($CV_{m}$)				
Repeatability Criterion (RC)	0%^a^	3%	6%	9%	12%
0.10 L/sec	0.00 L/sec 0.0%	0.08 L/sec 2.1%	0.18 L/sec 4.9%	0.30 L/sec 8.1%	0.41 L/sec 11%
0.15 L/sec	0.00 L/sec 0.0%	0.08 L/sec 2.1%	0.19 L/sec 5.1%	0.31 L/sec 8.4%	0.42 L/sec 11%
0.20 L/sec	0.00 L/sec 0.0%	0.07 L/sec 2.0%	0.07 L/sec 4.7%	0.28 L/sec 7.6%	0.40 L/sec 11%

*Notes*: Default subject characteristics from Table III. Intertest coefficient of variation ($CV^{t}$) = 3%. L/sec values reported ± 0.005 L/sec. Percentage values reported as two significant figures. Interquartile range: 25th−75th percentile of simulated distribution.

a Because 0% yields an undefined result, 0.01% is used to approximate the 0% value implicitly assumed in the ATS/ERS protocol and published studies.

## 5 DISCUSSION

RC discard some signals as if they were noise, and early test termination prevents the collection of potentially important signals. When inter‐ and intra‐test variabilities are assumed not to exist, all calculated pulmonary function changes are implicitly assumed to be real, not test protocol artifacts. This leads to unsupportable inferences about the statistical significance of observed differences. Measurement error alone could easily be greater.

Additional problems arise if tests are conducted only before and after exposure because intertest variability will not be accounted for. Guidelines recommend against drawing inferences from just two tests: “It is more likely that a real change has occurred when *more than two measurements* [i.e., tests] are performed over time” (Pellegrino, Viegi et al., 2005, p. 961, emphasis added). For the default comparison, any pair of test values must differ by more than 0.57 L/sec (16%) to be able to infer at *p* ≤ 0.05 that they are not drawn from the same distribution. A reasonable rule of thumb may be to refrain from interpreting as statistically meaningful any observed difference less than this amount unless and until inter‐ and intratest variability have been taken into account, both in data collection and statistical analysis.

### 5.1 Strengths and Limitations

Our analysis has several key strengths. First, we rely on widely accepted, peer‐reviewed studies of normal pulmonary function for all model parameters except for $CV_{m}$, for which the available literature is limited. Second, we infer a default value for intratest variability from NHANES, the “gold standard” for empirical data about the U.S. population. This inference is based on an examination of NHANES’ data exclusion rates, recognizing the similarity between the NHANES and ATS/ERS protocols. Third, our Monte Carlo model imposes no additional assumptions besides normality across and within tests for a single person. These assumptions can be modified to conduct unlimited sensitivity analyses.

Our analysis has many of the same limitations that affect most research in this field. Spirometry has other sources of inter‐ and intratest variability and potential bias, few of which typically are adequately controlled. Inter‐ and intratest variability can arise from technician quality (all technicians cannot be above average, much less superior), differences in spirometric devices (precision and accuracy vary), data entry, subject–device interactions, test settings, seasonal and diurnal effects, time periods between tests, and confounding effects. Indeed, the ATS/ERS protocols are commendable for including numerous elements intended to reduce the influence of confounders (ATS, 1979, 1987, 1995; Beydon et al., 2007a; Miller, Hankinson et al., 2005; Stocks et al., 2014).

Our simulation model has a related limitation insofar as it does not account for improvements in subject performance across maneuvers due to learning or decrements in subject performance across maneuvers due to fatigue. We are unable to capture this effect because no data appear to be publicly available. This is affected by coaching, the quality of which is variable and difficult to measure. It is intuitively reasonable to expect there is an optimal number of maneuvers where the gains from practice equal the losses from fatigue. But the optimum would vary across subjects, coaches, and other factors that cannot be easily modeled.

Intratest variability poses additional challenges. It may vary across subjects due to a host of factors. The period between maneuvers (not just tests) may matter, and the optimal spacing of maneuvers is both unknown and likely to vary across subjects. Finally, biological instability may arise between maneuvers insofar as testing induces rapid changes in lung volume that affect airway properties (Beydon et al., 2007a).

Our results assume that within‐person FEV_1_ is approximately normally distributed across both simulated independent tests and simulated independent maneuvers within each test. Results would differ with other distributional forms. We are aware of no empirical evidence supporting normality or any alternative distributional form. Normality across maneuvers might be refuted and could be informed by better intratest data collection, but we are aware of no way to theoretically inform the choice of the intratest distribution. At this stage of knowledge, it is more important to be transparent about the choice of distribution and cognizant of its potential significance. The effects of that choice cannot be quantified, however, as the number of alternative assumptions is infinitely large.

### 5.2 Practical Recommendations

The failure to account for intratest variability is a material limitation of conventional spirometry in research settings. There appears to have been no systematic effort to collect sufficient data to estimate intratest variability, whether for the population, research samples, or subpopulations of interest. All spirometric protocols recognize that intratest variability is important; hence, the universal guidance to conduct multiple maneuvers. But this recognition is abandoned in practice by terminating tests early, thus failing to collect needed data, and discarding all but a single fixed value to represent each test. The result is measurement error and bias.

Measurement error has pernicious effects on research intended to make causal inferences about small changes after treatment or exposure. A constructive path forward is to collect enough maneuver data to estimate $CV_{m}$ for the general population (e.g., NHANES), subpopulations presumed to be at greater risk (e.g., COPD patients, asthmatics, children), and any convenience sample (e.g., chamber study volunteers). Wherever possible, sample‐specific $CV_{m}$ should be estimated and tested against these reference values to ensure that inferences about the significance of observed changes are statistically valid.

## References

[risa13315-bib-0001] Adams, W. C. (2006a). Comparison of chamber 6.6‐hr exposures to 0.04‐0.08 ppm ozone via square‐wave and triangular profiles on pulmonary responses. Inhalation Toxicology, 18, 127–136. 16393927 10.1080/08958370500306107

[risa13315-bib-0002] Adams, W. C. (2006b). Human pulmonary responses with 30‐minute time intervals of exercise and rest when exposed for 8 hours to 0.12 ppm ozone via square‐wave and acute triangular profiles. Inhalation Toxicology, 18, 413–422. 16556581 10.1080/08958370600563599

[risa13315-bib-0003] American College of Occupational and Envirionmental Physicians . (2016). *Methodology for ACOEM's Occupational Medicine Practice Guidelines—2016 revision* . Elk Grove Village, IL: ACOEM.

[risa13315-bib-0004] American Thoracic Society [ATS] . (1979). ATS statement—Snowbird workshop on standardization of spirometry. American Review of Respiratory Disease, 119, 831–838. 453705 10.1164/arrd.1979.119.5.831

[risa13315-bib-0005] American Thoracic Society [ATS] . (1987). Standardization of spirometry: 1987 update. American Review of Respiratory Disease, 136, 1285–1298. 3674589 10.1164/ajrccm/136.5.1285

[risa13315-bib-0006] American Thoracic Society [ATS] . (1991). Lung function testing: Selection of reference values and interpretative strategies. American Review of Respiratory Disease, 144, 1202–1218. 1952453 10.1164/ajrccm/144.5.1202

[risa13315-bib-0007] American Thoracic Society [ATS] . (1995). Standardization of spirometry, 1994 update. American Journal of Respiratory and Critical Care Medicine, 152, 1107–1136. 7663792 10.1164/ajrccm.152.3.7663792

[risa13315-bib-0008] Aurora, P. , Stocks, J. , Oliver, C. , Sunders, C. , Castle, R. , Chaziparasidis, G. , & Bush, A. (2004). Quality control for spirometry in preschool children with and without lung disease. American Journal of Respiratory and Critical Care Medicine, 169(10), 1152–1159. 15028561 10.1164/rccm.200310-1453OC

[risa13315-bib-0009] Beydon, N. , Davis, S. D. , Lombardi, E. , Allen, J. L. , Arets, H. G. M. , Aurora, P. , … Eigen, H. (2007a). An official American Thoracic Society/European Respiratory Society statement: Pulmonary function testing in preschool children. American Journal of Respiratory and Critical Care Medicine, 175(12), 1304–1345. 17545458 10.1164/rccm.200605-642ST

[risa13315-bib-0010] Beydon, N. , Davis, S. D. , Lombardi, E. , Allen, J. L. , Arets, H. G. M. , Aurora, P. , … Eigen, H. (2007b). An official American Thoracic Society/European Respiratory Society statement: Pulmonary function testing in preschool children: Online supplementary materials. American Journal of Respiratory and Critical Care Medicine, 175(12), 1–206. 17545458 10.1164/rccm.200605-642ST

[risa13315-bib-0011] Brändli, O. , Schindler, C. , Künzli, N. , Keller, R. , & Perruchoud, A. (1996). Lung function in healthy never smoking adults: Reference values and lower limits of normal of a Swiss population. Thorax, 51(3), 277–283. 8779131 10.1136/thx.51.3.277 PMC1090639

[risa13315-bib-0012] Brown, J. S. (2007). *The effects of ozone on lung function at 0.06 ppm in healthy adults*. Research Triangle Park, NC: U.S. Environmental Protection Agency National Center for Environmental Assessment (EPA‐HQ‐OAR‐2005‐0172‐0175).

[risa13315-bib-0014] Enright, P. L. (2003). How to make sure your spirometry tests are of good quality. Respiratory Care, 48(8), 773–776. 12890297

[risa13315-bib-0015] Enright, P. L. , Beck, K. C. , & Sherrill, D. L. (2004). Repeatability of spirometry in 18,000 adult patients. American Journal of Respiratory and Critical Care Medicine, 169(2), 235–238. 14604836 10.1164/rccm.200204-347OC

[risa13315-bib-0016] Enright, P. L. , Johnson, L. R. , Connett, J. E. , Voelker, H. , & Buist, A. S. (1991). Spirometry in the Lung Health Study: 1. Methods and quality control. American Review of Respiratory Disease, 143(6), 1215–1223. 2048803 10.1164/ajrccm/143.6.1215

[risa13315-bib-0017] Enright, P. L. , Skloot, G. S. , Cox‐Ganser, J. M. , Udasin, I. G. , & Herbert, R. (2010). Quality of spirometry performed by 13,599 participants in the World Trade Center worker and volunteer medical screening program. Respiratory Care, 55(3), 303–309. 20196879

[risa13315-bib-0013] Enright, P. , Vollmer, W. , Lamprecht, B. , Jensen, R. , Jithoo, A. , Tan, W. , … Buist, A. S. (2011). Quality of spirometry tests performed by 9893 adults in 14 countries: The BOLD Study. Respiratory Medicine, 105(10), 1507–1515. 21549584 10.1016/j.rmed.2011.04.008

[risa13315-bib-0018] Hnizdo, E. , Glindmeyer, H. , & Petsonk, E. L. (2010). Workplace spirometry monitoring for respiratory disease prevention: A methods review. International Journal of Tuberculosis and Lung Disease, 14(7), 796–805. 20550761

[risa13315-bib-0019] Hnizdo, E. , Sircar, K. , Yan, T. , Harber, P. , Fleming, J. , & Glindmeyer, H. W. (2007). Limits of longitudinal decline for the interpretation of annual changes in FEV_1_ in individuals. Occupational and Environmental Medicine, 64(10), 701–707. 17478573 10.1136/oem.2006.031146 PMC2078388

[risa13315-bib-0020] Kainu, A. (2008). *Spirometric studies on the adult general population of Helsinki: Bronchodilation responses, determinants, and intrasession repeatability of FEV_1_, FEV_6_, FVC, and forced expiratory time* (Ph.D. thesis, University of Helsinki, Helsinki). Retrieved from https://helda.helsinki.fi/bitstream/handle/10138/22682/spiromet.pdf?sequence=1.

[risa13315-bib-0021] Kainu, A. , Lindqvist, A. , Sarna, S. , Lundbäck, B. , & Sovijärvi, A. (2008). FEV_1_ response to bronchodilation in an adult urban population. Chest, 134(2), 387–393. 18403671 10.1378/chest.07-2207

[risa13315-bib-0022] Kainu, A. , Lindqvist, A. , Sarna, S. , & Sovijärvi, A. (2008). Intra‐session repeatability of FET and FEV_6_ in the general population. Clinical Physiology and Functional Imaging, 28(3), 196–201. 18355346 10.1111/j.1475-097X.2008.00792.x

[risa13315-bib-0023] Künzli, N. , Ackermann‐Liebrich, U. , Brandli, O. , Tschopp, J. , Schindler, C. , & Leuenberger, P. (2000). Clinically “small” effects of air pollution on FVC have a large public health impact. Swiss Study on Air Pollution and Lung Disease in Adults (SAPALDIA)‐team. European Respiratory Journal, 15(1), 131–136. 10678634 10.1183/09031936.00.15113100

[risa13315-bib-0024] Künzli, N. , Ackermann‐Liebrich, U. , Keller, R. , Perruchoud, A. P. , & Schindler, C. (1995). Variability of FVC and FEV_1_ due to technician, team, device and subject in an eight centre study. European Respiratory Journal, 8, 371–376. 7789479 10.1183/09031936.95.08030371

[risa13315-bib-0025] Langhammer, A. , Johnsen, R. , Gulsvik, A. , Holmen, T. , & Bjermer, L. (2001). Forced spirometry reference values for Norwegian adults: The Bronchial Obstruction in Nord‐Trøndelag study. European Respiratory Journal, 18, 770–779. 11757626 10.1183/09031936.01.00255301

[risa13315-bib-0026] Lehmann, S. , Vollset, S. E. , Nygaard, H. A. , & Gulsvik, A. (2004). Factors determining performance of bronchodilator reversibility tests in middle‐aged and elderly. Respiratory Medicine, 98(11), 1071–1079. 15526807 10.1016/j.rmed.2004.03.019

[risa13315-bib-0027] Loeb, J. S. , Blower, W. C. , Feldstein, J. F. , Koch, B. A. , Munlin, A. L. , & Hardie, W. D. (2008). Acceptability and repeatability of spirometry in children using updated ATS/ERS criteria. Pediatric Pulmonology, 43(10), 1020–1024. 18785259 10.1002/ppul.20908

[risa13315-bib-0028] Miller, M. R. , Crapo, R. , Hankinson, J. , Brusasco, V. , Burgos, F. , Casaburi, R. , … Wanger, J. (2005). General considerations for lung function testing. European Respiratory Journal, 26(1), 153–161. 15994402 10.1183/09031936.05.00034505

[risa13315-bib-0029] Miller, M. R. , Hankinson, J. , Brusasco, V. , Burgos, F. , Casaburi, R. , Coates, A. , … Wanger, J. (2005). Standardisation of spirometry. European Respiratory Journal, 26(2), 319–338. 16055882 10.1183/09031936.05.00034805

[risa13315-bib-0030] National Health and Nutrition Examination Survey [NHANES] . (2008). Respiratory health: Spirometry procedures manual. Atlanta, GA: Centers for Disease Control.

[risa13315-bib-0031] National Health and Nutrition Examination Survey [NHANES] . (2011). *NHANES 2009–2010; Data documentation, codebook, and frequencies; Spirometry—1st test and 2nd test bronchodilator studies* . Retrieved from http://wwwn.cdc.gov/Nchs/Nhanes/2009-2010/SPX_F.XPT.

[risa13315-bib-0032] Pellegrino, R. , Decramer, M. , van Schayck, C. P. O. , Dekhuijzen, P. N. R. , Troosters, T. , van Herwaarden, C. , … Ardia, A. (2005). Quality control of spirometry: A lesson from the BRONCUS trial. European Respiratory Journal, 26(6), 1104–1109. 16319343 10.1183/09031936.05.00026705

[risa13315-bib-0033] Pellegrino, R. , Viegi, G. , Brusasco, V. , Crapo, R. O. , Burgos, F. , Casaburi, R. , … Wanger, J. (2005). Interpretative strategies for lung function tests. European Respiratory Journal, 26, 948–968. 16264058 10.1183/09031936.05.00035205

[risa13315-bib-0034] Redlich, C. A. , Tarlo, S. M. , Hankinson, J. L. , Townsend, M. C. , Eschenbacher, W. L. , Von Essen, S. G. , … Weissman, D. N. (2014). Official American Thoracic Society Technical Standards: Spirometry in the occupational setting. American Journal of Respiratory and Critical Care Medicine, 189(8), 984–994. 10.1164/rccm.201402-0337ST 24735032

[risa13315-bib-0035] Robey, R. R. , & Barcikowski, R. S. (1992). Type I error and the number of iterations in Monte Carlo studies of robustness. British Journal of Mathematical and Statistical Psychology, 45(2), 283–288.

[risa13315-bib-0036] Schelegle, E. S. , Morales, C. A. , Walby, W. F. , Marion, S. , & Allen, R. P. (2009). 6.6‐hour inhalation of ozone concentrations from 60 to 87 parts per billion in healthy humans. American Journal of Respiratory and Critical Care Medicine, 180(3), 265–272. 19447899 10.1164/rccm.200809-1484OC

[risa13315-bib-0037] Stocks, J. , Kirkby, J. , & Lum, S. (2014). How to avoid misinterpreting lung function tests in children: A few practical tips. Paediatric Respiratory Reviews, 15(2), 170–180. 24679988 10.1016/j.prrv.2014.02.001

[risa13315-bib-0038] U.S. Environmental Protection Agency [USEPA] . (2008). National Ambient Air Quality Standards for Ozone; Final Rule. Federal Register, 73(60), 16436–16514.

[risa13315-bib-0039] U.S. Environmental Protection Agency [USEPA] . (2015). National Ambient Air Quality Standards for Ozone; Final Rule. Federal Register, 80(206), 65292–65468.

